# Capturing Latino Health Disparities: Lessons from Mail- and Community-Based Population Health Surveys in California

**DOI:** 10.1158/2767-9764.CRC-25-0540

**Published:** 2026-04-20

**Authors:** April P. Vang, Juanita Elizabeth Quino, Angelica M. Rolon, Alexa Morales Arana, Julie H.T. Dang, Moon S. Chen, Primo N. Lara, Laura Fejerman, Luis G. Carvajal-Carmona

**Affiliations:** 1 The Health Equity Science and Community Research Laboratory (THE LCC Lab), Genome Center, University of California, Davis, Davis, California.; 2 University of California Davis Comprehensive Cancer Center, Sacramento, California.; 3 Department of Public Health Sciences, University of California, Davis, Davis, California.; 4 Department of Biochemistry and Molecular Medicine, School of Medicine, University of California, Davis, Davis, California.; 5 Office for Advancing Mentoring and the Professoriate, Inclusive Excellence Division, University of California, Davis, Davis, California.

## Abstract

Latinos face significant cancer health disparities and remain underrepresented in research. To address regional needs, the University of California Davis Comprehensive Cancer Center (UCDCCC) conducted its first Catchment Area Population Assessment (CAPA) to inform outreach and engagement strategies. Our purpose was to understand the best approaches for capturing Latino health need responses and to understand breast and cervical cancer screening rates and risk factors. This cross-sectional study compared two CAPA survey methodologies used from July 2019 to March 2020: a mail-based approach using random and unweighted probabilistic sampling of residential addresses versus a community-based approach using bilingual coordinators for in-person interviews. Comparisons were made on catchment population representativeness and breast and cervical cancer screening outcomes. The study included 361 Latino participants (255 community based and 106 mail based) across 16 of 19 UCDCCC catchment counties. Mail-based participants were significantly older, more educated, and had higher household incomes than community-based participants. Despite the differences in key demographics of participants in the two survey modalities, our results suggest that up-to-date breast and cervical cancer screening rates do not significantly differ. Time since the last routine checkup, primarily among community-based participants, emerged as a key predictor of screening adherence. Mail-based methods captured more affluent Latinos, whereas community-based methods reached more underserved populations. Each method introduced distinct sampling biases, but together they provided a more balanced representation of the catchment area. Mail- and community-based survey methods enhance the representativeness of Latino health data and support more equitable research recruitment strategies.

**Significance:**

Latinos remain underrepresented in cancer research, limiting effective outreach. This study demonstrates that combining mail- and community-based survey methods improves representation across socioeconomic groups, revealing key predictors of breast and cervical cancer screening adherence. These findings inform equitable data collection and engagement strategies, advancing inclusive cancer prevention efforts in underserved populations.

## Introduction

Latinos in the United States experience more infection-related cancers, such as stomach, liver, cervical tumors, and also tend to have higher rates of late-stage tumors of screenable cancers (1). They also have an increasing burden of obesity-related and early-onset tumors (2–7). These disparities are primarily driven by systemic and socioenvironmental inequities compounded by persistent underrepresentation in research, which limits the development of culturally responsive interventions and policies. Efforts to assess Latino community health needs are often hindered by mistrust in academic institutions and healthcare systems, as well as structural barriers to participation (8). To address these challenges, academic researchers must adopt inclusive and community-centered approaches that foster trust and engagement.

**Figure 1.**
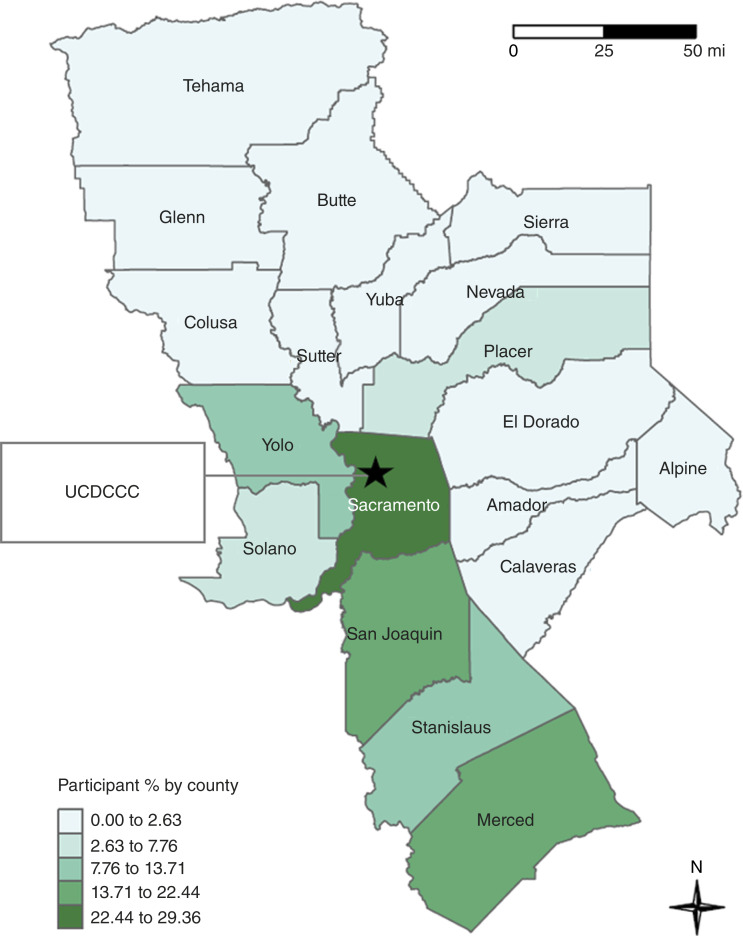
Participant recruitment by 19-county UCDCCC catchment area. Darker green indicates more participant inclusion from that county. Color ranges are according to hierarchical clustering.

In 2019, the University of California Davis Comprehensive Cancer Center (UCDCCC), the only NCI-designated cancer center serving the Central Valley and inland Northern California, launched a Catchment Area Population Assessment (CAPA) to better understand cancer-related needs throughout its 19-county region (Fig. 1). In partnership with Westat Inc., a company with data collection expertise, UCDCCC implemented a mail-based CAPA survey using probabilistic sampling to reach residents across the catchment area. The decision to use traditional mail surveys for gathering epidemiologic data in cancer health research was influenced by the rural character of the UCDCCC’s catchment area, which complicates outreach to the Latino population. Furthermore, it was assumed that participants would find it more convenient to complete the CAPA from home, resulting in more thorough responses. Concurrently, the Latinos United for Cancer Health Advancement (LUCHA) initiative was launched to specifically address cancer disparities among Latinos. LUCHA conducted a parallel CAPA survey (9) using community-based outreach, engaging bilingual and bicultural coordinators to administer in-person interviews at trusted community venues. In contrast to the mail-based approach, LUCHA’s efforts focused on community outreach and engagement, guided by the belief that healthcare professionals should approach the community rather than wait for it to seek them out. This framework is guided by the principle that, to gain the community’s trust, researchers and healthcare professionals need to be visible to its members, putting a face on those who wish to collaborate with them. Additionally, past studies indicate that community health fairs have been used to address unmet healthcare needs through low- or no-cost services focused on prevention and education in underserved communities.

Cancer is the second leading cause of death among US Latinos (7). Breast cancer is the most frequently diagnosed cancer and the leading cause of cancer death among Latinas (15%). Cervical cancer incidence is 15% to 36% higher in Latinas compared with non-Latino White females (7). Lower educational attainment, higher poverty rates, and limited access to preventive care exacerbate these disparities (7). Understanding screening behaviors, particularly for preventable malignancies such as breast and cervical cancers, is essential to improving early detection and reducing mortality in Latino populations.

This study compares two distinct CAPA survey methodologies, mail based versus community based, to evaluate their effectiveness in capturing representative data on Latino health needs. Specifically, our aims were (i) to assess which survey method more accurately reflects the sociodemographic characteristics of Latinos in the UCDCCC catchment area and (ii) to measure up-to-date breast and cervical cancer screening prevalence and identify associated risk factors. Insights from this comparison can inform future CAPA efforts across the 57 NCI-designated comprehensive cancer centers and guide equitable recruitment strategies for Latino populations.

## Materials and Methods

### Study design and recruitment

This cross-sectional study compared two survey methodologies used by the UCDCCC to assess Latino health needs across its 19-county catchment area between July 2019 and March 2020. The mail-based survey, developed in collaboration with Westat Inc., used an address-based, unweighted probabilistic sampling design to distribute 10,000 surveys, including 2,000 bilingual mailings targeting Latino households. The target was approximately 1,000 completed surveys to support regional estimates and subgroup comparisons. Sampled addresses were selected from the U.S. Postal Service Delivery Sequence File maintained by Marketing Systems Group and contacted via a two-stage mail protocol, consisting of an initial survey packet, followed by a reminder postcard mailed 4 to 5 business days later. To enhance inclusivity, surveys were translated into Spanish, and bilingual materials were sent to addresses matched to Hispanic surnames or located in census tracts with high concentrations of Hispanic residents. Spanish surveys were available upon request. The sample was drawn proportionally across the 19-county region; however, to improve representation of smaller counties, a supplemental sample of 100 addresses was later fielded, with 25 additional surveys mailed to each of four low-responding counties (Alpine, Amador, Colusa, and Sierra). Concurrently, the LUCHA initiative conducted a community-based survey using bilingual, bicultural staff at health fairs, churches, clinics, and other trusted venues. By partnering with key stakeholders in the area, including federally qualified health centers, UC Davis’s student-run clinics such as Clinica Tepati and the Knights Landing One Health Center, and the Mexican Consulate, among others, LUCHA set its goal of reaching the most vulnerable populations. LUCHA administered the survey both in person and online using the Qualtrics Research Suite. The surveys were shared with community networks and local contacts. For in-person administration, bilingual and bicultural research coordinators attended community events and locations, including health and wellness fairs, local churches, family centers, local agencies (such as the Mexican Consulate), and community clinics. The research team was available to provide verbal and written assistance with consent and survey completion. Participants could also scan a QR code to take the survey online. Because of the COVID-19 pandemic, LUCHA’s in-person efforts were halted, prompting the team to shift survey collection online, relying heavily on their extensive network of community partners, listservs, online classes, and social media. In total, the LUCHA team attended 17 in-person community events and successfully gathered 204 in-person surveys and 51 online surveys.

The studies involving human participants were reviewed and approved by UC Davis Institutional Review Board (IRB) and adhered to the U.S. Common Rule. We followed the UC Davis Investigator Manual HRP-103 for community-based participant recruitment. All participant studies were conducted in accordance with the recognized ethical guidelines. All research personnel involved in the study were fully trained in IRB human research subject protections. The patients/participants provided their written informed consent to participate in this study.

### Survey instrument

Both surveys (Supplementary Appendix S1–S4) were adapted from validated instruments, including the NCI Population Health Assessment Supplement (10) and the National Health Interview Survey (RRID: SCR_026761). The mail-based version included 48 questions; the community-based version had 64 questions. A total of 31 questions overlapped, and 25 were used for comparative analysis, covering sociodemographics, cancer screening, vaccination, health behaviors, and access (Supplementary Appendix S5). The mail-based survey included a standard set of items designed for the broader population, whereas the in-person, community-based survey included additional items specifically addressing cancer-related health disparities affecting Latino populations, such as colorectal cancer screening. As a result, the community-based survey was longer and more targeted, which may influence engagement differently. The tailored content may increase relevance and participation among Latino respondents, whereas the shorter, more general mail-based survey is expected to yield higher response rates although with fewer disparity-specific details.

### Eligibility criteria

Participants were adults (≥18 years) residing in the UCDCCC catchment area who self-identified as Latino. Mail-based participants were identified through address-based sampling; community-based participants were screened in person or online.

### Statistical analysis

We utilized the American Community Survey (ACS) 2016 to 2020 (RRID: SCR_024727) to obtain what we consider the gold-standard estimates of our target population, adult Latinos residing in our 19-county catchment area. Education attainment is among those 25 years of age and older and health insurance is among those 19 years of age and older. Medians are weighted by the percent of Latinos per county for ACS 2016 to 2020. We examine sociodemographic characteristics of Latinos, comparing community-based, mail-based, and community + mail estimates with the ACS 2016 to 2020 estimates. We use one-sample hypothesis testing with the proportion *z* test, Wilcoxon signed-rank test, and *χ*^2^ test where appropriate and consider differences at a two-sided *P* < 0.05 significance level. We plot counties by the proportion of participation in our study.

Furthermore, we compared responses by survey method or by up-to-date cancer screening status using *χ*^2^, Fisher exact, proportion, Wilcoxon, or *t* test where appropriate. These analyses are exploratory and descriptive given the nature of the project. We then used logistic regression models in the combined community + mail groups to identify predictors of being up-to-date with breast and cervical cancer screening. Age, education, and health insurance were considered confounding variables *a priori*. We also performed a supplemental within-survey analysis by up-to-date cancer screening status. We considered two-sided *P* < 0.05 to be significant. Model performance was evaluated using the Akaike information criterion, Hosmer–Lemeshow test, Linktest, and AUROC. Analyses were conducted using SAS v9.4 and R v4.2.2.

## Results

### Catchment area Latino representation

The UCDCCC catchment area covers 19 counties, with 30% of residents identifying as Latino. Supplementary Figure S1 shows the geographic distribution of Latino residents throughout the UCDCCC catchment area. A total of 835 responses were obtained from the 10,000 mailed surveys, of whom 106 (12.7%) self-identified as Latino. A total of 307 community-based survey entries were collected; however, only 255 were retained for analysis. Discarded community-based surveys included attempted survey entries captured by Qualtrics or in person that did not meet eligibility criteria, such as ethnicity (*n* = 2) or residence outside the catchment area (*n* = 18). This occurred due to two reasons. For online entries, Qualtrics retained all survey attempts, even when participants did not meet eligibility requirements and were unable to continue. For in-person surveys, if a coordinator did not assist a participant with the survey, the participant sometimes overlooked eligibility criteria and returned the survey. Surveys with completion rates below 33% (completed only through demographics; *n* = 32) were also excluded.

Our sample overall included 361 (*n* = 106 mail based and *n* = 255 community based) participants. We have at least one participant from 16 of the 19 counties. Shown in Fig. 1 and Supplementary Table S1, most of our participants live in the counties of Sacramento (29.4%), Merced (15.5%), San Joaquin (15%), Stanislaus (12.5%), and Yolo (11.6%). We captured a sample population which was from counties with higher densities of Latinos (Supplementary Fig. S1). We did not have Latino participants in three counties (Alpine, Nevada, and Sierra), which have lower population densities of Latinos in the east of our catchment counties (Fig. 1).

### Participant demographic and health characteristics

As shown in Table 1 for the 19-county catchment area, Latinos have a median age of 28 years and a median income of $60,000; 49.4% were female, 31.1% had less than a high school diploma, and 87.9% had health insurance. Both community- and mail-based approaches underrepresented those with less than a high school diploma (25% vs. 11.3% vs. 31.1%) and overrepresented those with a bachelor’s degree or higher (25% vs. 33% vs. 12.8%) compared with the ACS estimates. Community-based approaches captured 64.8% of those with household income below $50,000, whereas mail-based approaches captured 35.9%.

**Table 1.** Latino characteristics by community based vs. mail based vs. ACS 2016–2020 gold standard in UCDCCC 19-county catchment area.

Characteristic	Community based *n* = 255	Mail based *n* = 106	Overall (community + mail) *n* = 361	ACS 2016–2020
% Female	78.2%	79.8%	78.7%	49.4%
Median age (years)	40 years	52 years	43 years	28 years
Household income	64.8% below $50k	35.9% below $50k	56.5% below $50k	%50 below $60k
% Education attainment	​	​	​	​
Less than HS diploma	25%	11.3%	20.9%	31.1%
High school graduate or equv.	23.4%	17%	21.4%	28.1%
Some college or associate’s deg.	26.6%	38.7%	30.3%	28%
Bachelor’s degree or higher	25%	33%	27.4%	12.8%
% Health insurance, yes	72.4%	(NS) 91.3%	78.2%	87.9%

We consider gold-standard estimates reflective of Latinos within the UCDCCC 19-county catchment area to be according to the ACS 2016–2020, of which education attainment is among those 25 years of age and older and health insurance is among those 19 years of age and older. Proportion or median estimates are shown. Medians are weighted by the percent of Latinos per county for ACS 2016–2020. Note that median household income was $60k according to ACS 2016–2020, and we show percentages of the closest survey income category cutoff at $50k. One-sample proportion *z* test, one-sample Wilcoxon signed-rank test, and one-sample *χ*^2^ test are used for *P* values. Note that household income is not tested as there are mismatched thresholds; all else are significant except (NS) indication.

Abbreviations: HS, high school; NS, not significant.

The sociodemographic and health characteristics of our sample are displayed in Table 2. A total of 83.8% identified as Mexican and nearly half (47.2%) spoke both English and Spanish at home. Most had some college education (57.7%), were employed (56.4%), and earned <$50,000 annually (56.5%). Furthermore, 61.3% had private insurance, 74.2% had a routine checkup within the past year, and 40.2% had received at least one dose of the Hepatitis B vaccine. Among parents, 67.6% reported human papillomavirus vaccination for children 9 to 17 years of age. The average body mass index (BMI) was 30.4 (SD = 8), and participants exercised 3.8 days/week (SD = 2.2). Most rated their health as “Good” (44%), and 70.3% had sought health information. A total of 12.3% reported a cancer diagnosis.

**Table 2.** Participant demographic and health characteristics and behaviors by survey method.

Variable	Overall *n* = 361	Community based *n* = 255	Mail based *n* = 106	*P*
Mean age (SD)	44.58 (15.78)	41.05 (14.02)	52.8 (16.63)	<0.0001
Origin	​	​	​	<0.0001
Mexican, Mexican American, and Chicano	300 (83.8%)	227 (90.1%)	73 (68.9%)	​
Another Hispanic/Latino origin	58 (16.2%)	25 (9.9%)	33 (31.1%)	​
Language spoken at home	​	​	​	<0.0001
English only	92 (25.8%)	42 (16.8%)	50 (47.2%)	​
English and Spanish	168 (47.2%)	158 (63.2%)	10 (9.4%)	​
Spanish only	96 (27%)	50 (20%)	46 (43.4%)	​
Occupation	​	​	​	<0.0001
Employed	198 (56.4%)	140 (57.1%)	58 (54.7%)	​
Unemployed	31 (8.8%)	27 (11%)	4 (3.8%)	​
Homemaker	47 (13.4%)	40 (16.3%)	7 (6.6%)	​
Student	26 (7.4%)	25 (10.2%)	1 (0.9%)	​
Retired or disabled	49 (14%)	13 (5.3%)	36 (34%)	​
Household income	​	​	​	<0.0001
$0–$19,999	72 (22.6%)	63 (27.8%)	9 (9.8%)	​
$20,000–$49,999	108 (33.9%)	84 (37%)	24 (26.1%)	​
$50,000–$74,999	57 (17.9%)	38 (16.7%)	19 (20.7%)	​
$75,000–$99,999	33 (10.3%)	20 (8.8%)	13 (14.1%)	​
$100,000–$200,000	49 (15.4%)	22 (9.7%)	27 (29.3%)	​
Health insurance	​	​	​	<0.0001
None	72 (21.8%)	63 (27.6%)	9 (8.7%)	​
Private	203 (61.3%)	124 (54.4%)	79 (76.7%)	​
Public/other	56 (16.9%)	41 (18%)	15 (14.6%)	​
Time since last routine checkup	​	​	​	0.0409
1 year or less	256 (74.2%)	169 (70.7%)	87 (82.1%)	​
1–2 years	45 (13%)	33 (13.8%)	12 (11.3%)	​
2+ years	44 (12.8%)	37 (15.5%)	7 (6.6%)	​
Opinion of English speaking for non-English speakers		​	​	0.0019
Very well	105 (44.1%)	71 (38.6%)	34 (63%)	​
Well	49 (20.6%)	38 (20.7%)	11 (20.4%)	​
Not well	70 (29.4%)	64 (34.8%)	6 (11.1%)	​
Not at all	14 (5.9%)	11 (6%)	3 (5.6%)	​
Ever received Hepatitis B vaccine	​	​	​	0.8442
Yes, at least one dose	133 (40.2%)	93 (40.4%)	40 (39.6%)	​
No doses	69 (20.8%)	46 (20%)	23 (22.8%)	​
Do not know	129 (39%)	91 (39.6%)	38 (37.6%)	​
Child received HPV vaccine	​	​	​	0.5091
Yes	73 (67.6%)	49 (65.3%)	24 (72.7%)	​
No	35 (32.4%)	26 (34.7%)	9 (27.3%)	​
BMI	30.42 (8)	30.04 (8.58)	30.59 (7.72)	0.3173
Days exercise in the past 7 days	3.8 (2.21)	3.54 (2.2)	3.91 (2.21)	0.2243
Opinion of general health	​	​	​	0.5341
Excellent	28 (8.2%)	19 (7.9%)	9 (8.7%)	​
Very good	65 (19%)	43 (17.9%)	22 (21.4%)	​
Good	151 (44%)	108 (45%)	43 (41.7%)	​
Fair	79 (23%)	53 (22.1%)	26 (25.2%)	​
Poor	20 (5.8%)	17 (7.1%)	3 (2.9%)	​
Looked into health information	​	​	​	0.0005
Yes	242 (70.3%)	154 (64.4%)	88 (83.8%)	​
No	102 (29.7%)	85 (35.6%)	17 (16.2%)	​
Cancer identified by health professional	​	​	​	0.1847
Yes	43 (12.3%)	26 (10.6%)	17 (16.3%)	​
No	307 (87.7%)	220 (89.4%)	87 (83.7%)	​

Abbreviation: HPV, human papillomavirus.

Two-sided *P* values reported from *χ*^2^, Fisher exact, or Wilcoxon test. Count and proportions are shown for categorical variables. Mean and SDs are shown for BMI and days of exercise in the past 7 days.

### Survey method comparisons

Mail-based participants were older (52.8 vs. 41.1 years; *P* < 0.01; Table 2), more educated (*P* < 0.04; Table 1), and had higher incomes (*P* < 0.01; Table 2). They were more likely to speak only English at home (*P* < 0.01; Table 2).

Occupationally, mail-based respondents were less likely to be unemployed, homemakers, or students and more likely to be retired or disabled (*P* < 0.01; Table 2).

Mail-based participants also had lower uninsured rates and higher private insurance coverage (*P* < 0.01; Table 2), more routine checkups within the year (*P* = 0.0409; Table 2), greater English proficiency (*P* < 0.01; Table 2), and were more likely to look into health information (83.3% vs. 64.4%; *P* < 0.01; Table 2). No differences were observed in BMI, exercise, or general health.

### Cancer screening prevalence and factors associated with screening

#### Breast

**Figure 2.**
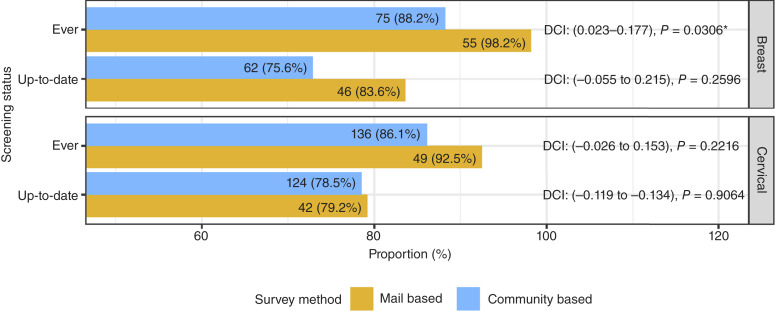
Prevalence of breast and cervical cancer screening of Latinas in the UCDCCC catchment counties by survey method, from July 2019 to March 2020. Cervical cancer screening: females ages 21–65 years (*n* = 211); up-to-date is within 3 years. There were 87.7% who ever had a Pap smear, and 78.7% were up-to-date. Breast cancer screening: females ages 40–75 years (*n* = 141); up-to-date is within 2 years. Note that there were four participants who did not specify the time since their last mammogram; this makes denominators for up-to-date mammogram *n* = 137 overall, *n* = 82 for community based, and *n* = 55 for mail based. There were 92.2% who ever had a mammogram, and 78.8% who were up-to-date. Count *n* and proportion (%) are shown. DCI, difference CI. CI is for the difference of mail-based proportion minus community-based proportion. Two-sided *P* values reported from equality of proportions test. *, *P* < 0.05.

During our survey period (July 2019–March 2020), the up-to-date status for breast cancer screening required receiving a mammogram within the recommended 2 years for women 40 to 75 years of age. Among *n* = 141 eligible Latinas (Fig. 2), 92.2% ever had a mammogram and 78.8% were up-to-date (within 2 years). Rates for *ever* receiving breast cancer screening significantly differed by survey modality (98.2% mail based vs. 88.2% community based, *P* = 0.03; Fig. 2) although we did not detect meaningful differences for being up-to-date for breast cancer screening. The up-to-date breast cancer screening rate for mail-based participants was between 5.5 percentage points lower and 21.5 percentage points higher than that of community-based participants at the 95% confidence level (Fig. 2). Shown in Supplementary Table S2 for breast cancer screening, up-to-date status was associated with having private health insurance (73.8% vs. 33.3%), a routine checkup within the year (81.5% vs. 50%; *P* < 0.01), no cancer diagnosis (73.8% vs. 92.6%; *P* = 0.04), and seeking health information (81.9% vs. 58.6%; *P* = 0.02). Mail-based participants tended to be more up-to-date with breast cancer screenings (42% up-to-date vs. 31% not up-to-date, Supplementary Table S2) although not significantly different (*P* = 0.37).

#### Cervical

During our survey period, the up-to-date status for cervical cancer screening required having a Pap smear within the recommended 3 years for women 21 to 65 years of age. Among *n* = 211 eligible Latinas (Fig. 2), 87.7% ever had a Pap smear, and 78.7% were up-to-date (within 3 years). Although cervical cancer screening rates for ever having (92.5% vs. 86.1%; Fig. 2) and up-to-date (79.2% vs. 78.5%; Fig. 2) were higher among mail-based participants, screening rates did not differ significantly. The up-to-date cervical cancer screening rate for mail-based participants was between 11.9 percentage points lower and 13.4 percentage points higher than that of community-based participants at the 95% confidence level, indicating no evidence of a difference between the two groups (Fig. 2). Being up-to-date with cervical screening was associated with younger age (49 vs. 54.8 years; *P* < 0.01), higher education (67.7% vs. 40.9%; *P* < 0.01), more recent routine checkup (75.3% vs. 50%; *P* < 0.01), and no cancer diagnosis (90.1% vs. 77.3%; *P* = 0.05). No major differences in up-to-date cervical screening rates were noted between mail-based and community-based participants (Supplementary Table S2).

### Multiple logistic regression models for up-to-date cervical and breast cancer screening

**Figure 3.**
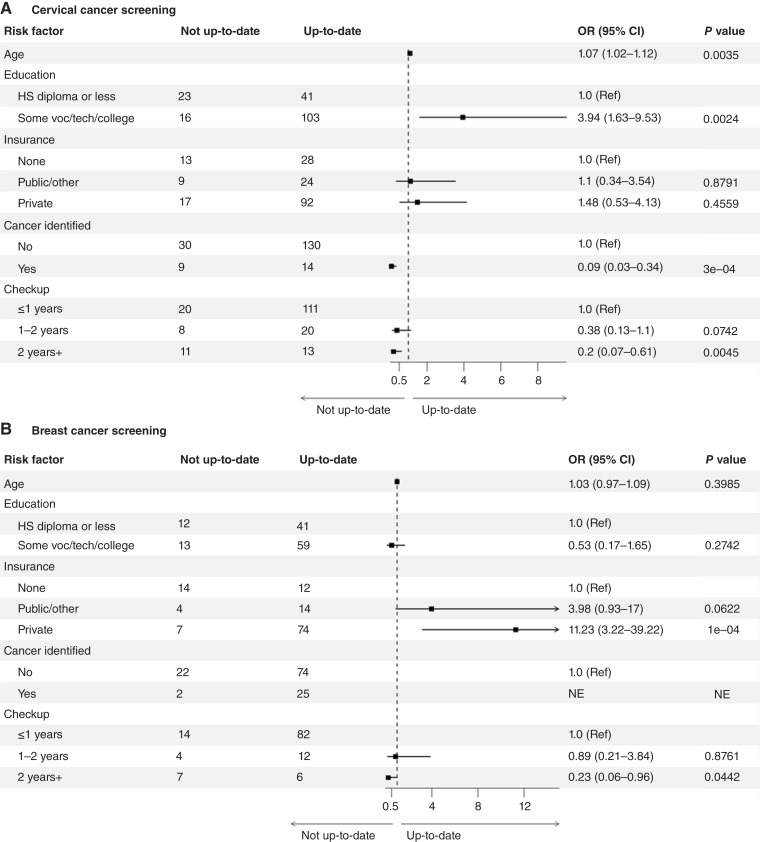
ORs for up-to-date status in cervical (**A**) and breast (**B**) cancer screening from multiple logistic regression. Only complete variables were included in modeling. NE indicates not estimated if variable was not selected under Akaike information criterion with forward stepwise logistic regression. Small model includes three variables—age, education, and health insurance. Full model includes nine variables—age, education, health insurance, survey type, BMI, last routine checkup, opinion of general health, looked into health information, and cancer identified by health professional. HS, high school; Ref, reference.

In adjusted models (Fig. 3A), Latinas with some college or vocational education had 3.94 times the odds of being up-to-date with cervical cancer screening [95% confidence interval (CI), 1.63–9.53; *P* < 0.01] compared with those with a high school diploma or less. Those with a routine checkup of more than 2 years ago had 80% lower odds (*P* < 0.01) compared with those with a routine checkup within the year, and those with any cancer diagnosis had 91% lower odds (*P* = 0.0003) compared with those with no cancer diagnosis for being up-to-date with cervical cancer screening.

For breast cancer screening (Fig. 3B), private insurance was associated with an 11.23-fold increase in the odds of being up to date (95% CI, 3.22–39.22; *P* < 0.01) compared with those with no health insurance. Having a routine checkup more than 2 years ago was associated with 77% lower odds (*P* < 0.05) of being up-to-date with breast cancer screening compared with those having a routine checkup within the year. Education was not a significant predictor (*P* = 0.27) for up-to-date breast cancer screening.

Both models are appropriately specified and yield 80% AUC (Supplementary Table S3). In our supplemental within-survey analysis, routine checkups continue to be important among community-based participants but were no longer associated with, or a significant predictor of, both cancer screenings among mail-based participants (Supplementary Table S2; Supplementary Fig. S2).

### Medical delays

Table 3 presents results on the reasons for delays when participants needed to see a doctor. Note that the mail-based survey asked participants to mark all reasons for a delay, whereas the community-based survey asked participants to mark only one primary delay. Overall, 28.5% of participants experienced delays, and the top reason for both mail- and community-based groups was that they could not get an appointment. Among community-based participants, the next top reasons for delays were that they did not have time, could not afford it, or did not have insurance. Among mail-based participants, the next top reasons for delays were that they did not have insurance, hours were not convenient, and they could not afford it or found it too expensive.

**Table 3.** Delay when needing to see a doctor by survey method.

​	Overall (*n* = 361)		Community based (*n* = 255)		Mail based (*n* = 106)	
	*n*	%	*n*	%	*n*	%
Delay when needing to see a doctor	​	​	​	​	​	​
No	233	64.5%	162	63.5%	71	67%
Yes	103	28.5%	70	27.5%	33	31.1%
Could not get appointment	27	7.5%	10	3.9%	17	16%
Insurance was not accepted	5	1.4%	4	1.6%	1	0.9%
Insurance did not cover	10	2.8%	9	3.5%	1	0.9%
Language problems	1	0.3%	0	0%	1	0.9%
Transportation problems	6	1.7%	2	0.8%	4	3.8%
Hours were not convenient	14	3.9%	6	2.4%	8	7.5%
No child care for children at home	2	0.6%	2	0.8%	0	0%
Did not have time	13	3.6%	7	2.7%	6	5.7%
Forgot or lost referral	3	0.8%	3	1.2%	—	—
Could not afford/cost too much	15	4.2%	7	2.7%	8	7.5%
No insurance	17	4.7%	7	2.7%	10	9.4%
Other	9	2.5%	5	2%	4	3.8%
Do not know/refuse/no response	8	2.2%	8	3.1%	0	0%
Do not know/refuse/no response	25	6.9%	23	9%	2	1.9%

Note that there were differences in surveys; the mail-based survey asked to mark all reasons if delayed whereas the community-based survey asked to mark only one main reason. “—” indicates category not listed as an option in mail-based survey. Other includes concern about symptoms, did not have the courage, have a headache, not an emergency, waiting for insurance approval for specialist, weather (snowing), physician not accepting new patients, office visits too much, and traveling to Mexico for cancer care instead of waiting for 3 months for appointment in the United States.

## Discussion

Understanding and addressing cancer disparities require accurate, community-informed data that reflect the lived experiences of diverse populations. CAPAs are a key part of this effort, enabling NCI-designated cancer centers to systematically assess the health needs, barriers, and behaviors of the populations they serve (11). For the UCDCCC, which covers a large and diverse region of inland Northern California, CAPA surveys are essential for guiding outreach, research, and policy initiatives tailored to Latino communities who continue to be disproportionately affected by cancer and are underrepresented in research. By comparing mail-based and community-based CAPA approaches, this study provides valuable insights into how survey design influences data representativeness and the identification of cancer screening gaps.

Although our initial goal was to identify the most effective survey method for collecting Latino health data, our findings suggest that a combined approach is most effective when resources are available. Each method targeted different segments of the population, and together they produced a sample that more accurately reflects the sociodemographic profile of Latinos in the UCDCCC catchment area, according to the 5-year ACS estimates for 2016 to 2020 (RRID: SCR_024727). For example, mail-based surveys mostly captured individuals with higher income, whereas community-based surveys were more successful in reaching lower-income participants. A similar trend was observed for occupational status: mail-based methods included more retired or disabled individuals, whereas community-based strategies engaged more unemployed and homemaker respondents. Our mail-based sample may disproportionately capture more affluent and English-speaking respondents because of greater housing stability, literacy, and familiarity with research participation. Individuals who are employed or have disabilities may also be more likely to respond because mail surveys can be completed at home and on a flexible schedule, reducing logistic and mobility barriers. Together, these factors likely contributed to nonresponse bias, limiting participation among lower-income, limited–English proficiency, and more structurally marginalized populations. Community-based recruitment had more restricted coverage because outreach was concentrated at specific sites and in counties with higher Latino populations. Given the geographic dispersion of Latino residents across rural and agricultural areas, individuals living in less targeted or more isolated counties were less likely to be reached. This focus narrowed the overall geographic coverage of the community-based approach.

We included an online survey panel to rapidly reach a large, geographically diverse population and help supplement underrepresented Latino groups. Opt-in panels (e.g., Dynata, Qualtrics, and Prolific) are cost-effective but may introduce selection bias as participants self-select and may not fully represent Latino populations, particularly those with limited internet access or lower English proficiency. Address-based sampling panels (e.g., NORC and Ipsos) use probabilistic selection to improve representativeness, but they may still miss segments of the Latino population, such as those who are mobile, live in rural or multi-unit housing, or face language and literacy barriers.

Although we ideally want a more effective way to randomly survey a representative sample of the catchment area, in the absence of such an approach, we, however, combined two methods that together help capture more of the diversity of the underlying population, which is better than using just one or the other. The mail-based approach, for example, disproportionately captured more affluent individuals, those with higher education, greater income, private insurance, and better English-speaking ability. It also had a low response rate of 5.3% (106 of 2,000 Latino-targeted addresses), raising concerns about nonresponse bias. Conversely, the community-based method was more effective at reaching underserved populations but was geographically limited, covering only 12 of the 19 counties in the catchment area (Supplementary Table S1), and it used convenience sampling. Both survey modalities introduce self-selection bias in which participants who respond are those who want to be surveyed and thus lead to nonrandom participation. In addition, both survey modalities overrepresented individuals with higher education to be included; 25% of community-based respondents had a bachelor’s degree or higher, almost twice the ACS estimate of 12.8%. The mail-based sample showed an even greater overrepresentation at 33%. The shift to online data collection during the COVID-19 pandemic may have further skewed the sample toward younger, more tech-savvy individuals, including university students, as 20% of community-based responses were collected online.

Mail-based participants showed a trend toward higher rates of being up-to-date with breast and cervical cancer screenings compared with community-based participants although these differences may be due to chance (Fig. 2). These findings suggest that community-based participants, despite representing a more underserved demographic, may be equally engaged in preventive care, potentially because of increased access and outreach efforts that bring healthcare services directly into trusted community settings.

Consistent with prior studies (12), our findings highlight the critical role of routine checkups in promoting adherence to breast and cervical cancer screening guidelines. Routine checkups, defined as preventive visits not prompted by illness, were strongly associated with screening status. Latinas who had not had a routine checkup in over 2 years had 77% lower odds of being up-to-date with breast cancer screening (*P* = 0.0442) and 80% lower odds of being up-to-date with cervical cancer screening (*P* = 0.0045). These findings suggest that routine engagement with the healthcare system may reflect greater health literacy and proactive health behaviors, including the ability to navigate insurance, schedule appointments, and communicate with providers. However, access barriers remain a concern. In our sample, 28.5% of participants reported delayed care when needed, most commonly because of difficulty securing appointments (Table 3). Participants expressed access to care, knowledge, and awareness concerns (Supplementary Table S4). This points to systemic issues such as provider shortages, long wait times, and scheduling inefficiencies that disproportionately affect underserved populations consistent with past findings (13). Addressing these barriers is essential to improving screening uptake and reducing disparities in cancer outcomes.

We acknowledge some limitations in our study. The sample sizes for our cancer screening models were limited by the requirement to include only female participants within guideline-recommended age ranges and with complete data. For cervical cancer screening, our model included 183 Latinas 21 to 65 years of age, whereas power calculations (using the R package *pmsampsize* for multivariable logistic regression, assuming a prevalence of 0.6, four predictors, a 0.9 overfit adjustment, and an AUROC of 0.8) indicate that 248 participants are needed to attain 80% power. Likewise, our breast cancer screening model included 125 Latinas 40 to 75 years of age, which is below the estimated requirement of 369 participants. Although both models showed acceptable performance, the limited sample size may have decreased the precision of effect estimates. These limitations highlight broader challenges in recruiting and retaining Latino participants in research and emphasize the need for ongoing, culturally sensitive engagement strategies to better understand the needs, voices, and barriers faced by this population.

Looking ahead, the UCDCCC aims to advance health equity through ongoing, community-engaged research. LUCHA, working with the Office of Outreach and Engagement, continues to address concerns from Latino participants, such as limited resource awareness, uncertainty about cancer prevention, fear of care, and preference for natural remedies. These highlight the need to co-develop strategies with the community to build trust, improve health literacy, and reduce barriers.

In conclusion, our study demonstrates that combining mail- and community-based survey methods enhances the representativeness of Latino health data and provides a more comprehensive understanding of cancer screening behaviors. This dual-method approach offers a practical model for other NCI-designated cancer centers seeking to conduct equitable catchment area assessments and develop responsive, community-informed cancer control strategies. We believe that the study results indicate that both methods are complementary; however, a community-based approach would be particularly informative in the post-pandemic period as several studies have shown that the pandemic led to lower screening rates in underserved communities. It would be important for NCI-designated cancer centers to assess whether screening rates in this community have returned to normal levels.

## Supplementary Material

Supplementary Table 1 Participant by county and survey method

Supplementary Table 2 Risk factors by current cervical and breast cancer screening

Supplementary Table 3 Post-regression analysis for current screening status

Supplementary Table 4 Feedback from participants by survey method

Appendix 1 Community - English Survey

Appendix 2 Community - Spanish Survey

Appendix 3 Mail - English Survey

Appendix 4 Mail - Spanish Survey

Appendix 5 Community-Mail-Merged-Survey Questions

## Data Availability

The raw, deidentified data supporting the conclusions of this article will be made available on reasonable request by the authors without undue reservation.
