# Supplementary material for: Capturing Latino Health Disparities: Lessons from Mail- and Community-Based Population Health Surveys in California
Source: Cancer Res Commun. 2026 Apr 20;6(4):873–83. doi: 10.1158/2767-9764.CRC-25-0540 (PMC13095202; doi:10.1158/2767-9764.CRC-25-0540)
Supplement: Appendix 1 — Community - English Survey [file crc-25-0540_appendix_1_supps1.pdf]

UC DAVIS HEALTH | COMPREHENSIVE CANCER CENTER

Latino Catchment Area Population Health Assessment

Consent

1. Welcome to the research study!   Hello! We are the University of California Davis Comprehensive Cancer Center. Our Latino Cancer Health Equity Initiative is conducting a research study to gather information about your health. To gather this information, we ask that you complete this survey. This survey should take less than 15 minutes to complete. However, completing the survey is completely voluntary. Voluntary means you can agree or not agree to complete it. It is up to you. You may also skip or refuse to answer questions you do not want to answer. But, we hope that you will answer all of them. Any information about who you are will also be kept secret. We will not be able to identify or share your name or ID number from your survey responses.

If you have any questions about the survey, just ask us!
LUCHA Director, Luis G. Carvajal-Carmona PHD
hs-iniciativalatina@ucdavis.edu | (916) 734-4563

By checking the circle below, you acknowledge that your participation in the study is voluntary, you are 18 years of age, and that you are aware that you may choose to terminate your participation in the study at any time and for any reason during the survey questionnaire.

Please mark one below:

- ☐ I consent, I wish to participate in this study
- ☐ I do not consent; I do not wish to participate in this study

Eligibility Pre-screening Questions

Please write in, mark or fill in the circle next to your answer for each question.

2. Are you at least 18 years of age?

- o Yes
- o No

3. Are you Hispanic or Latino?

- o Yes
- o No

4. What is your race/ethnicity? One or more categories may be selected. Mark all that apply.

- o South American
- o Central American
- o Mexican
- o Dominican Republic
- o Puerto Rican
- o Cuban
- o Other: ________________
- o Don't know
- o Refuse to answer

5. What county do you live in?

- o Alpine
- o Amador
- o Butte
- o Calaveras
- o Colusa
- o El Dorado
- o Glenn
- o Merced
- o Nevada
- o Placer
- o Sacramento
- o San Joaquin
- o Sierra
- o Solano
- o Stanislaus
- o Sutter
- o Tehama
- o Yolo
- o Yuba
- o Other

6. What is your zip code?

____

7. What year were you born?

____

Survey Questionnaire

8. Are you male or female?

- o Male
- o Female
- o Don’t know
- o Refuse to answer

9. What is the highest grade or level of schooling you completed?

- o Less than 8 years
- o 8 through 11 years
- o 12 years or completed high school
- o Post high school training other than college (vocational or technical)
- o Some college
- o College graduate
- o Postgraduate
- o Don't know
- o Refuse to answer

10. What language do you speak at home?

- o English (skip to #12)
- o Spanish
- o Other (specify: ___________________________)
- o Don't know
- o Refuse to answer

11. Since you speak a language other than English at home, we are interested in your own opinion of how well you speak English. Would you say you speak English?

- o Very well
- o Well
- o Not well
- o Not at all
- o Don’t know
- o Refuse to answer

12. Thinking about members of your family living in this household, what is your combined annual income, meaning the total pre-tax income from all sources earned in the past year?

- o Less than $10,000
- o 10,000 to under $15,000
- o $15,000 to under $20,000
- o $20,000 to under $35,000
- o $35,000 to under $50,000
- o $50,000 to under $75,000
- o $75,000 to under $100,000
- o $100,000 to under $200,000
- o $200,000 or more
- o Don't know/not sure
- o Refuse to answer

13. Do you have any kind of health care coverage, including health insurance, prepaid plans such as HMOs, government plans such as Medicare, or Indian Health Service?

o Yes o No o Don't know/ not sure o Refuse to answer

14. If you answered yes, what type of health care coverage do you have?

- o A plan purchased through an employer or union (includes plans purchased through another person's employer)
- o A plan that you or another family member buys on your own
- o Medicare
- o Medicaid or other state program
- o Tricare (formerly CHAMPUS), VA, or Military
- o Alaska Native, Indian Health Service, Tribal Health Services
- o Some other source
- o None (no coverage)
- o Don't know/not sure
- o Refuse to answer

15. What is your current occupational status? Mark only one.

- o Employed, full time
- o Employed, part time
- o Unemployed
- o Homemaker
- o Student
- o Self Employed
- o Retired
- o Disabled
- o Other________
- o Don't know
- o Refuse to answer

16. Have you ever looked for information about health or medical topics from any source?

o Yes o No o Don't know o Refuse to answer

17. If yes, the most recent time you looked for information about health or medical topics, where did you go first?

- o Books
- o Brochures, pamphlets, etc.
- o Cancer organization
- o Family
- o Friend/coworker
- o Doctor or health care provider
- o Internet
- o Library
- o Magazines
- o Newspapers
- o Telephone information number
- o Complementary, alternative, or unconventional practitioner
- o Social media site, such as Facebook, patientslikeme, caring bridge
- o Other ___________________
- o Don't know
- o Refuse to answer

18. Overall how confident are you that you could get advice or information about health or medical topics if you needed it?

- o Completely confident
- o Very confident
- o Somewhat confident
- o A little confident
- o Not confident at all
- o Don't know
- o Refuse to answer

19. What is the first hospital that comes to mind when you think about cancer care?

____

20. Before this survey, have you ever heard of the University of California Davis, and of its Comprehensive Cancer Center / Cancer Institute?

- Yes
- No
- Don't know
- Refuse to answer

21. Is there a specific social media site you like to go to for health or medical information?

- Yes
- No
- Don't know
- Refuse to answer

22. If you answered yes, what social media site do you go to for health or medical information?

- Facebook
- Twitter
- Instagram
- LinkedIn
- Pinterest
- Snapchat
- Other: ___________
- Don't know
- Refuse to answer

23. Are you 49 years of age or older?

- Yes
- No (skip to #28)

24. The next questions are about colorectal cancer screening.
A Colonoscopy is an exam in which a tube is inserted in the rectum to view the colon for signs of cancer or other health problems. Have you ever had this exam?

- Yes
- No
- Don't know
- Refuse to answer

25. If you answered yes, when was the last time you had a colonoscopy?

- Within the past 10 years
- More than 10 or more years ago
- Don't know/not sure
- Refuse to answer

26. A blood stool test is a test that may use a special kit at home to determine whether the stool contains blood (like Fecal Occult Blood Test (FOBT), a Fecal Immunochemical Test (FIT) or Cologuard). Have you ever had this test using a home kit?

- Yes
- No
- Don't know
- Refuse to answer

27. If you answered yes, how long has it been since you had you last blood stool test using a home kit?

- Within the past year (anytime less than 12 months ago)
- Within the past 2 years (1 year but less than 2 years ago)
- Within the past 3 years (2 years but less than 3 years ago)
- Within the past 5 years (3 years but less than 5 years ago)
- 5 or more years ago
- Don't know/not sure
- Refuse to answer

28. The next questions are about cervical cancer. *(If you are not a female, skip to question #32)*
A pap exam is a procedure to test for cervical cancer in women. A pap smear involves collecting cells from your cervix-the lower, narrow end of your uterus that's at the top of your vagina. Have you ever had a pap smear?

- Yes
- No
- Don't know/ not sure
- Refuse to answer

29. If yes, how long has it been since you had your last pap smear?

- o Within the past year (anytime less than 12 months ago)
- o Within the past 2 years (1 year but less than 2 years ago)
- o Within the past 3 years (2 years but less than 3 years ago)
- o Within the past 5 years (3 years but less than 5 years ago)
- o 5 or more years ago
- o Don't know/not sure
- o Refuse to answer

30. The next questions are about breast cancer. A mammogram is an x-ray of each breast to look for breast cancer. Have you ever had a mammogram?

o Yes o No o Don't know/ not sure o Refuse to answer

31. If yes, how long has it been since you had your last mammogram?

- o Within the past year (anytime less than 12 months ago)
- o Within the past 2 years (1 year but less than 2 years ago)
- o Within the past 3 years (2 years but less than 3 years ago)
- o Within the past 5 years (3 years but less than 5 years ago)
- o 5 or more years ago
- o Don't know/not sure
- o Refuse to answer

32. The next question is intended for parents of children ages 9-17. Do you have children of age of 9-17?

o Yes o No (skip to #34)

33. Human Papillomavirus (HPV) vaccine is given to prevent HPV related cancers and genital warts. The HPV vaccines available are called Cervarix, Gardasil or Gardasil 9. It is given in 2 or 3 separate doses over a 6-month period. Has your child ever received one or more doses of the HPV vaccine?

o Yes o No o Don't know o Refuse to answer

34. Hepatitis B Vaccine is given in three separate doses and has been recommended for all newborn infants since 1991. In 1995, it was recommended that adolescents be given the vaccine. Persons who may be exposed to other people's blood, such as health care workers, also may have received the vaccine. Have you ever received the 3 dose series of the Hepatitis B Vaccine?

- o Yes at least 3 doses
- o Yes, less than 3 doses
- o No doses
- o Don't know
- o Refuse to answer

35. Have you smoked at least 100 cigarettes in your entire life?

o Yes o No o Don't know o Refuse to answer

36. If yes, do you now smoke cigarettes…

- o Every day
- o Some days
- o Not at all
- o Don't know
- o Refuse to answer

37. Considering all types of alcoholic beverages, how many times during the past 30 days did you have x [men = 5, women = 4] or more drinks on an occasion?

- Number of times: ______
- None
- Don't know/ not sure
- Refuse to answer

38. Now think about the foods you ate during the past month that is, the past 30 days, including meals and snacks. During the past month, how many times did you eat fruit? Do not count juices. You can tell us per day, per week, or month.

- __________times per day
- __________times week
- __________times month
- Don't know
- Refuse to answer

39. Now think about the foods you ate during the past month that is, the past 30 days, including meals and snacks. During the past month, how many times did you eat any other vegetables like green salad, green beans or potatoes? Do not include fried potatoes.

- __________times per day
- __________times week
- __________times month
- Don't know
- Refuse to answer

40. The next question is about your overall exercise. Exercise can include walking, housekeeping, construction, hard manual labor, field work, jogging, weights, a sport or playing with your kids. It can be done on the job, around the house, just for fun or as a work-out. In the past 7 days, on how many days did you exercise for at least 20 minutes at a time?

- ______ days per week
- Don't know
- Refuse to answer

41. How tall are you without shoes?

- ________feet/________inches
- ________meters /________centimeters
- Don't know
- Refuse to answer

42. How much do you weight without shoes?

- __________pounds
- __________kilograms
- Don't know
- Refuse to answer

43. About how long has it been since you last saw a doctor or medical provider for a routine check-up? [if needed: a routine check-up is a visit not for an illness or problem. This visit may include questions about health behaviors such as smoking.]

- One year ago, or less
- More than 1 up to 2 years ago
- More than 2 up to 5 years ago
- More than 5 years ago
- Never
- Don't know
- Refuse to answer

44. Would you say that in general your health is excellent, very good, good, fair, or poor?

- Excellent
- Very good
- Good
- Fair
- Poor
- Don't know
- Refuse to answer

45. Is there a place that you usually go to when you are sick or need advice about your health?

- Yes
- There is no place
- There is more than one place
- Don't know/not sure
- Refuse to answer

46. If yes, what kind of place do you go most often?

- Clinic or health center
- Doctor's office or HMO
- Hospital emergency room
- Hospital outpatient department
- Some other place
- Doesn't go to one place most often
- Don't know/not sure
- Refuse to answer

47. During the past 12 months, did you delay or not get any other medical care you felt you needed—such as seeing a doctor, a specialist, or other health professional?

- Yes
- No
- Don't know
- Refuse to answer

48. If yes, what was the one main reason why you delayed getting the care you felt you needed?

- o Couldn't get appointment
- o My insurance not accepted
- o Insurance did not cover
- o Language problems
- o Transportation problems
- o Hours not convenient
- o No child care for children at home
- o Forgot or lost referral
- o I didn't have time
- o Couldn't afford/cost too much
- o No insurance
- o Other (specify: ____________)
- o Don't know
- o Refuse to answer

49. Have you ever been told by a doctor or other health professional that you had cancer or a malignancy of any kind?

o Yes o No (skip to #52) o Don't know o Refuse to answer

50. If yes, what type of cancer was it?

____

51. Since your diagnosis, has anyone discussed with you whether you would like to take part in cancer research? If yes, did you participate and in what?

- o Clinical trials
- o Biospecimen donation
- o Other: _______________________________________
- o Don't Know
- o Refuse to answer

52. What do you think are the most important cancer health problems in your community?

____
____
____
____

53. Do you have a family history of cancer?

o Yes o No (skip to #54) o Don't know o Refuse to answer

If yes, what type of cancers have affected your family?

____

54. What media do you use to learn about advances in health, medicine, cancer care?

- o Social media
- o Email
- o Mail
- o Newspaper
- o Radio
- o TV
- o Don't Know
- o Refuse to answer

55. If you chose TV, in what language do you see the programs?

- o English
- o Spanish
- o Other______________
- o Don't know
- o Refuse to answer

56. What Country were you born in?

- o Country: _____________
- o Don't know
- o Refuse to answer

57. How many years have you been living in the United States?

- o Years: ____________
- o Don't Know
- o Refuse to answer

58. Do you travel to Mexico or another country to see a doctor?

o Yes   o No   o Don't know   o Refuse to answer

59. Have you ever been diagnosed with one of the following? You can select more than one.

- o Helicobacter Pylori Infection
- o Gastritis
- o Gastric Ulcer
- o Gastro-Esophageal Reflux (GERD)
- o Any other stomach problem: _____________________
- o No (skip to #62)
- o Don't Know
- o Refuse to answer

60. Did you receive treatment for the condition above? (Helicobacter pylori, Gastric Ulcer, Gastritis, Gastro-Esophageal Reflux)

o Yes   o No (skip to #62)   o Don't know   o Refuse to answer

61. If yes, what was the treatment received for the condition?

____

62. Have you ever been diagnosed with one of the following?

- o Liver Cirrhosis
- o Hepatitis B
- o Hepatitis C
- o Non-Alcoholic Fatty Liver (NAFL)
- o Non-Alcoholic Steatohepatitis (NASH)
- o Other Liver Disease: ____________________
- o No (skip to the end)
- o Don't Know
- o Refuse to answer

Continue to the end→

63. Did you receive treatment for the condition above? (Liver Cirrhosis, Hepatitis B, Hepatitis C, Non-Alcoholic Fatty Liver or Non-Alcoholic Steatohepatitis)

- o Yes
- o No
- o Don't know
- o Refuse to answer

64. If yes, what was the treatment?

____

Please feel free to leave a comment on the survey, cancer, your community needs etc

____

____

____

____

____

____
