# Supplementary material for: Capturing Latino Health Disparities: Lessons from Mail- and Community-Based Population Health Surveys in California
Source: Cancer Res Commun. 2026 Apr 20;6(4):873–83. doi: 10.1158/2767-9764.CRC-25-0540 (PMC13095202; doi:10.1158/2767-9764.CRC-25-0540)
Supplement: Appendix 2 — Community - Spanish Survey [file crc-25-0540_appendix_2_supps2.pdf]

UCDAVIS HEALTH | COMPREHENSIVE CANCER CENTER

Encuesta de Evaluación de Salud en la Comunidad Latina

Consentimiento

1. ¡Hola! Somos la Universidad de California Davis Centro Integral de Cáncer. Nuestra Iniciativa de Equidad en la Salud del Cáncer Latino está llevando a cabo un estudio de investigación para colectar información sobre su salud. Para colectar esta información, le pedimos que complete esta encuesta. Esta encuesta tomara menos de 15 minutos para completar. Completar la encuesta es completamente voluntario. Voluntario significa que puede estar de acuerdo o no en completarlo. Es tu decisión. También puede omitir o Prefiero no responder las preguntas que no quiera responder. Pero, esperamos que usted responda a todas. Cualquier información sobre quién eres también se mantendrá en secreto. No podemos identificar su nombre o número de identificación con sus respuestas en esta encuesta.

¡Si tiene alguna pregunta sobre la encuesta, solo pregúntenos!
LUCHA Director, Luis G Carvajal- Carmona PHD
hs-iniciativalatina@ucdavis.edu | (916) 734-4563

Al marcar el circulo de abajo, reconoce que su participación en el estudio es voluntaria, tiene 18 años de edad y que es consciente de que puede cancelar su participación en el estudio en cualquier momento y por cualquier motivo durante la encuesta.

Por favor marcar uno:

- ☐ **Doy mi consentimiento, si deseo participar en el estudio**
- ☐ **No doy mi consentimiento, no deseo participar en el estudio**

Preguntas de Elegibilidad

Por favor escriba, marque o llene el círculo al lado de su respuesta para cada pregunta.

1. ¿Tienes al menos 18 años de edad?
- ○ Si
- ○ No

2. ¿Es hispano/a o Latino/a?
- ○ Si
- ○ No

3. ¿Cuál es su raza/origen étnico? Se pueden seleccionar una o más categorías. Marque todos los que apliquen.
- ○ Sur Americano/a
- ○ Centro Americano/a
- ○ Mexicano/a
- ○ Dominicano/a
- ○ Puertorriqueño/a
- ○ Cubano/a
- ○ Otro: _______________
- ○ No sé
- ○ Prefiero no responder

5. ¿En que condado vives?

○ Alpine	○ Sacramento
○ Amador	○ San Joaquin
○ Butte	○ Sierra
○ Calaveras	○ Solano
○ Colusa	○ Stanislaus
○ El Dorado	○ Sutter
○ Glenn	○ Tehama
○ Merced	○ Yolo
○ Nevada	○ Yuba
○ Placer	○ Otro

6. ¿Cuál es su código postal?

____

7. ¿En qué año nació?

____

Encuesta

8. Sexo: ¿Eres hombre o mujer?

- Hombre
- Mujer
- No sé
- Prefiero no responder

9. ¿Cuál es el grado más alto o nivel de escuela que has completado?

- Menos de 8 años
- 8 a 11 años
- 12 años o completó la escuela secundaria
- Entrenamiento de la escuela secundaria que no sea la Universidad (vocacional o técnica)
- Alguna universidad
- Graduado universitario
- Estudios de posgrado
- No sé
- Prefiero no responder

10. ¿Qué idioma hablas en casa?

- Inglés (siga a pregunta #12)
- Español
- Otro (especifica: _______________)
- No sé
- Prefiero no responder

11. Porque usted habla otro idioma que ingles en casa, estamos interesados en su propia opinión de lo bien que usted habla inglés. Usted diría que hablas ingles

- Muy bien
- Bien
- No muy bien
- Absolutamente nada
- No sé
- Prefiero no responder

12. Pensando en todos los miembros de su familia que viven en su hogar, ¿cuál es su ingreso combinado anualmente? Esto quiere decir, el total de ingresos antes de impuestos de todas las fuentes obtenidas en el año pasado. El total combinado es

- Menos de $10,000
- $10,000 a menos de $15,000
- $15,000 a menos de $20,000
- $20,000 a menos de $35,000
- $35,000 a menos de $50,000
- $50,000 a menos de $75,000
- $75,000 a menos de $100,000
- $100,000 a menos de $200,000
- $200,000 o mas
- No sé/ no estoy seguro
- Prefiero no responder

13. ¿Tiene algún tipo de cobertura de salud, incluyendo seguro de salud, planes prepagados como HMOs, planes de gobierno como Medicare o el Servicio de Salud de Indio?

- Si
- No
- No sé
- Prefiero no responder

14. Si contesto si, ¿qué tipo de cobertura de salud tiene?

- Un plan comprado a través de un empleador o unión (incluye planes comprados a través del empleador de otra persona)
- Un plan que usted u otro miembro de la familia compro por su cuenta
- Medicare
- Medicaid u otro programa de gobierno
- TRICARE (Anteriormente CHAMPUS), VA, o Militaría
- Nativo de Alaska, servicio de salud indio, servicios de salud tribal
- Alguna otra fuente
- Ninguna (sin cobertura)
- No sé /No estoy seguro
- Prefiero no responder

15. ¿Cuál es su estado profesional en este momento? Marca sólo uno.

- Empleado, tiempo completo
- Empleado, medio tiempo
- Desempleados
- Casero
- Estudiante
- Trabajador por cuenta propia
- Jubilado
- Deshabilitado
- Otro: _______________
- No sé
- Prefiero no responder

16. ¿Alguna vez ha buscado información sobre temas médicos o de salud de alguna fuente?

- Si
- No
- No sé / no estoy seguro
- Prefiero no responder

17. Si contestó sí, ¿Cuál fue la última vez que buscó información sobre temas médicos o de salud, a dónde fue primero?

- Libros
- Folletos
- Organización de Cáncer
- Familia
- Amigo/ compañero
- Doctor o Proveedor de Atención Médica
- Internet
- Biblioteca
- Revistas
- Periódicos
- Número de Información Telefónica
- Profesional Complementario, Alternativo, o no Convencional
- Sitio de medios sociales, como Facebook, PatientsLikeMe, Caring Bridge, Pacientes Como Yo, Puente de Cuidado
- Otro: ______________
- No sé
- Prefiero no responder

18. En general, ¿Qué tan confiado es usted en poder obtener consejos o información sobre temas médicos o de salud si lo necesitara?

- Completamente Confiado
- Muy Confiado
- Algo Confiado
- Un Poco Confiado
- Sin Confianza
- No sé
- Prefiero no responder

19. ¿Cuál es el primer hospital que viene a la mente cuando piensas en el cuidado de cáncer?

____

20. Antes de esta encuesta, ¿alguna vez ha oído hablar de la Universidad de California Davis, y de su Centro Integral de Cáncer/Instituto de Cáncer?

- Si
- No
- No sé
- Prefiero no responder

21. ¿Hay un sitio de redes sociales especifico al que le guste acudir para obtener información médica o de salud?

- Si
- No
- No sé
- Prefiero no responder

22. Si respondió sí, ¿qué sitio de rede social utilizas para obtener información de salud o medica?

- Facebook
- Twitter
- Instagram
- LinkedIn
- Pinterest
- Snapchat
- Otro: ____________
- No sé
- Prefiero no responder

23. ¿Tiene 49 años o más?

- Si
- No (siga a pregunta #28)

24. Las siguientes preguntas son sobre la detección del cáncer colorrecta *(también llamado cáncer de colon)*. Una colonoscopia es un examen en el cual se inserta un tubo en el recto para ver el colon en busca de síntomas de cáncer u otros problemas de salud. ¿Alguna vez has tenido este examen?

- Si
- No
- No sé
- Prefiero no responder

25. Si respondió sí, ¿cuánto tiempo ha pasado desde que tuvo su último examen de colonoscopia?

- En los últimos 10 años
- Mas de 10 años
- No sé/ no estoy seguro
- Prefiero no responder

26. Una prueba de heces de sangre es una prueba que puede usar un kit especial en el hogar para determinar si las heces contienen sangre (como la prueba de sangre oculta en heces (FOBT), la prueba inmunoquímica fecal (FIT) o Cologuard). ¿Alguna vez te has hecho esta prueba con un kit especial en casa?

- Si
- No
- No sé
- Prefiero no responder

27. Si respondió sí, ¿cuánto tiempo ha pasado desde que se hizo su último análisis de heces con un kit para el hogar?

- En él último año (en cualquier momento menos de 12 meses atrás)
- En los últimos 2 años (hace 1 año, pero menos de 2 años)
- En los últimos 3 años (hace 2 años, pero menos de 3 años)
- En los últimos 5 años (hace 3 años, pero menos de 5 años)
- Hace 5 o más años
- No sé /No estoy seguro
- Prefiero no responder

28. Las siguientes preguntas son sobre el cáncer cervical o de cuello uterino. (*Si no es mujer, siga a pregunta #32)* Un examen de Pap es un procedimiento para examinar el cáncer cervical en mujeres. Una Papanicolau consiste en colectar células del cuello uterino, al extremo inferior y estrecho del útero que está en la parte superior de la vagina, ¿alguna vez tuviste una prueba de Papanicolaou?

- Si
- No
- No sé
- Prefiero no responder

29. Si respondió sí, ¿cuánto tiempo ha pasado desde que tuvo su última prueba de Papanicolau?

- En él último año (en cualquier momento menos de 12 meses atrás)
- En los últimos 2 años (hace 1 año, pero menos de 2 años)
- En los últimos 3 años (hace 2 años, pero menos de 3 años)
- En los últimos 5 años (hace 3 años, pero menos de 5 años)
- Hace 5 años o más
- No Sé/ no estoy seguro
- Prefiero no responder

30. Las siguientes preguntas son sobre el cáncer de seno. Una mamografía es una radiografía de cada seno para buscar cáncer de seno, ¿alguna vez te has hecho una mamografía?

- Si
- No
- No sé
- Prefiero no responder

31. Si respondió sí, ¿cuánto tiempo ha pasado desde que tuvo su última mamografía?

- En él último año (en cualquier momento menos de 12 meses atrás)
- En los últimos 2 años (hace 1 año, pero menos de 2 años)
- En los últimos 3 años (hace 2 años, pero menos de 3 años)
- En los últimos 5 años (hace 3 años, pero menos de 5 años)
- Hace 5 años o más
- No sé /No estoy seguro
- Prefiero no responder

32. Las siguientes preguntas están dirigidas a los padres de niños de 9 a 17 años. ¿Tienes hijos de 9 a 17 años?

- Si
- No (siga a pregunta #34)

33. La vacuna contra el Virus del Papiloma Humano (VPH) se administra para prevenir los cánceres relacionados con el VPH y las verrugas genitales. Las vacunas contra el VPH son disponibles y se llaman Cervarix, Gardasil o Gardasil 9. Se administra en 2 o 3 dosis separadas durante un periodo de 6 meses, ¿su hijo/a ha recibido alguna vez una o más dosis de la vacuna contra el VPH?

- Si
- No
- No sé
- Prefiero no responder

34. La vacuna contra la Hepatitis B se administra en tres dosis separadas y se ha recomendado para todos los bebés recién nacidos desde 1991. En 1995, se recomendó administrar la vacuna a los adolescentes. Las personas que puedan haber estado expuestas a la sangre de otras personas, como los trabajadores sanitarios, también pueden haber recibido la vacuna, ¿ha recibido alguna vez la serie de 3 doses de la vacuna contra la hepatitis B?

- Si, al menos 3 dosis
- Si, menos de 3 dosis
- No
- No sé
- Prefiero no responder

35. ¿Has fumado al menos 100 cigarrillos en toda tu vida?

- Si
- No (siga a #37)
- No sé
- Prefiero no responder

36. Si respondió sí, en este momento usted fuma cigarrillos…

- Todos los días
- Algunos días
- Nunca
- No sé
- Prefiero no responder

37. Teniendo en cuenta todos los tipos de bebidas alcohólicas, ¿Cuántas veces durante los últimos 30 días tuviste (Hombres: 5, Mujeres: 4) o más bebidas en una ocasión?

- Número de veces: __________
- Nada
- No sé
- Prefiero no responder

38. Ahora piense en los alimentos que comió durante el mes pasado, es decir, los últimos 30 días, incluyendo las comidas y aperitivos. Durante el mes pasado, ¿Cuántas veces comió fruta? No cuente los jugos. ¿Puede decírmelo por día, por semana o por mes?

- ___________ veces por día
- ___________ veces por semana
- ___________ veces por mes
- No sé
- Prefiero no responder

39. Ahora piense en los alimentos que comió durante el mes pasado, es decir, los últimos 30 días, incluyendo las comidas y aperitivos. Durante el mes pasado, ¿Cuántas veces comió otros vegetales como ensalada verde, ejotes o papas? (No incluyen papas fritas) Puede decírmelo por día, por semana o mes.

- ___________ veces por día
- ___________ veces por semana
- ___________ veces por mes
- No sé
- Prefiero no responder

40. La siguiente pregunta es acerca de su ejercicio general. El ejercicio puede incluir actividades como caminar, limpieza del hogar, trotear, pesas, un deporte o jugar con sus hijos. Se puede hacer en el trabajo, alrededor de la casa, sólo por diversión o como un entrenamiento. En los últimos 7 días, ¿cuántos días hizo ejercicio por lo menos 20 minutos a la vez?

- ___________ días por semana
- No sé
- Prefiero no responder

41. Las siguientes preguntas son sobre su estatura y peso, ¿cuál es su altura sin zapatos?

- Pies________ Pulgadas: ______
- Metros________Centímetros: ______
- No sé
- Prefiero no responder

42. ¿Cuánto pesa sin zapatos?

- ___________ libras
- ______________kilos
- No sé
- Prefiero no responder

43. ¿Cuánto tiempo ha pasado desde la última vez que vio a un doctor o proveedor médico para un chequeo de rutina? [si es necesario: un chequeo de rutina es una visita, no por una enfermedad o problema. Esta visita puede incluir preguntas sobre comportamientos de salud como fumar.]

- Hace un año o menos
- Entre 1 y 2 años
- Entre 2 y 5 años
- Hace más de 5 años
- Nunca
- No sé
- Prefiero no responder

44. ¿Dirías que en general tu salud es excelente, muy buena, buena, justa o pobre?

- Excelente
- Muy buena
- Buena
- Justa
- Pobre
- No sé
- Prefiero no responder

45. ¿Hay algún lugar al que usted usualmente va cuando está enfermo o necesita consejo sobre su salud?

- Si
- No hay lugar
- Hay más que un lugar
- No sé/ no estoy seguro
- Prefiero no responder

46. Si contesto sí, ¿qué tipo de lugar vas más frecuente?

- Clínica o Centro de Salud
- Consultorio Médico o HMO
- Sala de Emergencias en el Hospital
- Centro Ambulatorio del Hospital
- Algún otro Lugar
- No va a un lugar con más frecuencia
- No sé/ no estoy seguro
- Prefiero no responder

47. Durante los últimos 12 meses, ¿te retrasaste o no recibiste ninguna otra atención médica que sentiste que necesitabas, como ver a un médico, un especialista u otro profesional de salud?

- Si
- No
- No sé
- Prefiero no responder

48. Si contesto sí, ¿finalmente recibió atención? Si es que si, ¿cuál fue la razón por la que retrasó recibir la atención medica que sintió necesitaba?

- No pude conseguir mi cita
- Mi seguro no es aceptado
- Mi seguro no lo cubre
- Problemas de idioma
- Problemas de transporte
- Horas no son convenientes
- Falta de guardería para niños
- Olvidó o perdió la referencia
- No tuve tiempo
- No podría pagar / cuesta demasiado
- No tuve seguro
- Otro: ____________
- No sé
- Prefiero no responder

49. ¿Alguna vez te ha dicho un médico u otro profesional de salud que tuviste cáncer o un tumor maligno de cualquier tipo?

- Si
- No (siga a #52)
- No sé
- Prefiero no responder

50. Si contesto sí, ¿qué tipo de cáncer era y a qué edad?

____

51. Desde su diagnóstico, ¿alguien ha hablado con usted si le gustaría participar en la investigación sobre el cáncer? Si es así, ¿en qué participaste?

- Ensayos Clínicos
- Donación de Muestras Biológicas
- Otro: __________________
- No se
- Prefiero no responder

52. ¿Cuáles crees que son los problemas de salud oncológicos (cáncer) más importantes en tu comunidad?

____

____

____

____

53. ¿Usted tiene historia familiar de cáncer?

- Si
- No (siga a #54)
- No sé
- Prefiero no responder

Si contesto si, ¿Qué tipo de canceres han afectado a su familia?

____

54. ¿Qué medios utilizas para aprender sobre los avances en salud, medicina, cuidado del cáncer?

- Sitio de medios sociales
- Correo electrónico
- Correo Postal
- Periódico
- Radio
- Televisión
- No se
- Prefiero no responder

55. ¿Si escogió televisión, en que idioma ve sus programas?

- Ingles
- Español
- Otro: ___________
- No se
- Prefiero no responder

56. ¿En qué país nació?

- País: _______________________________________
- No se
- Prefiero no responder

57. ¿Cuántos años lleva viviendo en Estados Unidos?

- Años: _____________
- No se
- Prefiero no responder

58. ¿Usted viaja a México o a otro país para ver a un doctor?

- Si
- No
- No sé
- Prefiero no responder

59. ¿Alguna vez lo han diagnosticado con una de las enfermedades siguientes? Puede seleccionar más que uno.

- Infección de Helicobacter pylori
- Gastritis
- Úlcera gástrica
- Reflujo gastroesofágico (GERD)
- Cualquier otro problema estomacal: ____________
- No (siga a #62)
- No se
- Prefiero no responder

60. ¿Recibió tratamiento para la condición anterior? (Helicobacter pylori, ulcera gástrica, gastritis o reflujo gastroesofágico)

- Si
- No
- No sé
- Prefiero no responder

61. Si contesto si, ¿cuál fue el tratamiento?

____

62. ¿Alguna vez le han diagnosticado uno de los siguientes?

- Cirrosis hepática
- Hepatitis B
- Hepatitis C
- Hígado graso no alcohólico (NAFL)
- Esteatohepatitis no alcohólica (NASH)
- Otras enfermedades del hígado: ________
- No (siga al final)
- No se
- Prefiero no responder

63. ¿Usted recibió tratamiento por la condición anterior? (cirrosis hepática, hepatitis B, hepatitis C, Hígado graso no alcohólico o Esteatohepatitis no alcohólica)

- Si
- No
- No sé
- Prefiero no responder

64. Si contesto si, ¿cuál fue el tratamiento?

____

Si gusta, deje comentario sobre la encuesta, cáncer, tu comunidad etc..

____
____
____
____
____
____
____
