# Supplementary material for: Capturing Latino Health Disparities: Lessons from Mail- and Community-Based Population Health Surveys in California
Source: Cancer Res Commun. 2026 Apr 20;6(4):873–83. doi: 10.1158/2767-9764.CRC-25-0540 (PMC13095202; doi:10.1158/2767-9764.CRC-25-0540)
Supplement: Appendix 3 — Mail - English Survey [file crc-25-0540_appendix_3_supps3.pdf]

University of California, Davis Comprehensive Cancer Center
Community Health Assessment

The purpose of this questionnaire is to learn how we might serve you and others in your community better in providing cancer prevention outreach, disease control, and education. We estimate that this will take about 10 minutes (or less).

Instructions:
- **Please use a black or blue pen to complete this form.**
- **Mark ☒ to indicate your answer. If you want to change your answer, darken the box ■ and mark the correct answer.**

1. Have you ever looked for information about health or medical topics from any source?

☐ Yes
☐ No → GO TO QUESTION 5.

2. To whom or where did you go first for information about health or medical topics on the most recent occasion?

☐ Doctor or health care provider
☐ Family
☐ Other people, e.g., friends, co-workers, advice telephone line
☐ Printed matter, e.g., books, magazines, pamphlets
☐ Social media including the internet
☐ Other

3. Is there a specific social media site you like to go to for health or medical information?

☐ Yes
☐ No → GO TO QUESTION 5.
☐ Don't know → GO TO QUESTION 5.

4. Which media platforms do you use for health or medical information? Mark all that apply.

☐ Facebook
☐ Twitter
☐ Instagram
☐ LinkedIn
☐ Pinterest
☐ Snapchat
☐ Other

5. What is the first hospital that comes to mind when you think about cancer care?

[ ]

6. Before this survey, had you ever heard of the University of California, Davis Comprehensive Cancer Center?

☐ Yes
☐ No

Cancer screening: Questions 7-11 are for women only.
Men, please go to Question 12.

7. A mammogram is an X-ray of each breast to look for breast cancer. Have you ever had a mammogram?

- ☐ Yes
- ☐ No → GO TO QUESTION 10.

8. How old were you when you had your first mammogram? ☐☐

9. How long has it been since you had your last mammogram?

- ☐ Within the past year (anytime less than 12 months ago)
- ☐ Within the past 2 years (at least 1 year but less than 2 years ago)
- ☐ Within the past 3 years (at least 2 years but less than 3 years ago)
- ☐ Within the past 5 years (at least 3 years but less than 5 years ago)
- ☐ 5 or more years ago
- ☐ Don't know

10. The next questions are about cervical cancer. A Pap smear is a procedure to test for cervical cancer in women. Have you ever had a Pap smear?

- ☐ Yes
- ☐ No → GO TO QUESTION 12.
- ☐ Don't know → GO TO QUESTION 12.

11. How long has it been since you had your last Pap smear?

- ☐ Within the past year (anytime less than 12 months ago)
- ☐ Within the past 2 years (at least 1 year but less than 2 years ago)
- ☐ Within the past 3 years (at least 2 years but less than 3 years ago)
- ☐ Within the past 5 years (at least 3 years but less than 5 years ago)
- ☐ 5 or more years ago

12. The Hepatitis B vaccine is given in three separate doses and has been recommended for all newborn infants since 1991. Have you ever received the 3-dose series of the Hepatitis B vaccine?

- ☐ Yes, the full series of either 2 or 3 doses
- ☐ Received at least one dose
- ☐ No doses (not vaccinated)
- ☐ Don't know

For parents of children currently ages 9-17. All others go to Question 15.

13. Have all your child(ren) aged 9-17 received one or more doses of the Human Papillomavirus (HPV) vaccine?

- ☐ Yes → GO TO QUESTION 15.
- ☐ No
- ☐ Don't know → GO TO QUESTION 15.

Draft

14. Which of the following explains your reason(s) for your child(ren) not being vaccinated for HPV? Mark all that apply.

- ☐ Don't know enough about the HPV vaccine
- ☐ Doctor did not recommend it
- ☐ Don't think the vaccine is needed
- ☐ Did not know the vaccine could prevent cancer
- ☐ Financial factors: too expensive or not covered by insurance
- ☐ Safety concerns with the vaccine
- ☐ Other, please specify: ________

Tobacco

15. What is your current smoking status? A Smoker is someone who used cigarettes in the past 30 days.

- ☐ Smoker → GO TO QUESTION 17.
- ☐ Former Smoker
- ☐ Never Smoker → GO TO QUESTION 20.

16. At what age did you quit smoking for the last time?

☐☐ Years old

17. How many total years have you smoked (or did you smoke) cigarettes? Do not count any time that you have stayed off cigarettes.

☐☐ Years

18. On average, when you have smoked, about how many cigarettes do you (or did you) smoke a day? A pack usually has 20 cigarettes in it.

☐☐ Number of cigarettes a day

19. Have you ever had a CT scan for lung cancer screening?

- ☐ Yes
- ☐ No

Exercise: The next question is about your overall exercise habits. Exercise includes walking, housekeeping, jogging, lifting weights, playing a sport, or playing with your kids. It can be done on the job, around the house, just for fun, or as a workout.

20. In the past 7 days, on how many days did you exercise for at least 20 minutes at a time?

☐ Days per week

- ☐ Don't know

Cancer beliefs

How much do you agree or disagree with each of the following statements?

	Strongly agree	Somewhat agree	Somewhat disagree	Strongly disagree	Don't know	Prefer not to answer
**21.** It seems like everything causes cancer.	☐	☐	☐	☐	☐	☐
**22.** There is not much you can do to lower your chances of getting cancer.	☐	☐	☐	☐	☐	☐
**23.** There are so many different recommendations about preventing cancer; it's hard to know which ones to follow.	☐	☐	☐	☐	☐	☐

Health care access

24. Where do you go to seek medical advice? Mark all that apply.

- ☐ Clinic or health center
- ☐ Doctor's office or HMO
- ☐ Hospital emergency room
- ☐ Hospital outpatient department
- ☐ Some other place

25. In the past 12 months, was there a time when you needed to see a doctor, but could not?

- ☐ No, I was able to see a doctor without any barriers → GO TO QUESTION 26.

MARK ALL OF THE FOLLOWING THAT APPLY:

- ☐ Yes, because I couldn't get an appointment
- ☐ Yes, because my insurance was not accepted
- ☐ Yes, because insurance did not cover
- ☐ Yes, because of language problems
- ☐ Yes, because of transportation problems
- ☐ Yes, because hours were not convenient
- ☐ Yes, because there was no child care for children at home
- ☐ Yes, because I didn't have time
- ☐ Yes, because I couldn't afford/cost too much
- ☐ Yes, because I had no insurance
- ☐ Other, please specify: ____________
- ☐ Don't know

26. Have you ever been told by a doctor or other health professional that you have or had cancer of any kind?

- ☐ Yes → What type(s) of cancer was it? ____________
- ☐ No → GO TO QUESTION 30.
- ☐ Don't know

Draft

27. How old were you when you were diagnosed? ☐☐

☐ Don't know

28. Since your cancer diagnosis, did your doctor ever recommend a clinical trial?

☐ Yes, but I did not choose to participate
☐ Yes, I participated in a clinical trial
☐ No, the doctor did not recommend a clinical trial for me
☐ Don't know or don't remember

29. Since your cancer diagnosis, did you donate any tissue, e.g., blood, cells, etc. for cancer research?

☐ Yes
☐ No, but I would be willing to do so
☐ No, and I don't want to do so
☐ Don't know

Health status: The next questions are about your overall health.

30. How tall are you without shoes?

☐☐ Feet ☐☐ Inches

☐ Don't know

31. How much do you weigh without shoes?

☐☐☐ Pounds

☐ Don't know

32. About how long has it been since you last saw a doctor or medical provider for a routine check-up?

☐ One year ago or less
☐ More than 1 up to 2 years ago
☐ More than 2 up to 5 years ago
☐ More than 5 years ago
☐ Never
☐ Don't know

33. Would you say that in general your health is excellent, very good, good, fair, or poor?

☐ Excellent
☐ Very Good
☐ Good
☐ Fair
☐ Poor
☐ Don't know

Draft

Personal characteristics

34. Are you male or female?

- ☐ Male
- ☐ Female

35. Are you of Hispanic, Latino/a, or Spanish origin? Mark all that apply.

- ☐ Mexican, Mexican American, Chicano/a
- ☐ Puerto Rican
- ☐ Cuban
- ☐ Another Hispanic, Latino/a, or Spanish origin
- ☐ None of these

36. Which is the racial group that you associate with? Mark all that apply.

- ☐ White
- ☐ Black or African American
- ☐ American Indian or Alaska Native
- ☐ Asian Indian
- ☐ Chinese
- ☐ Filipino
- ☐ Hmong
- ☐ Japanese
- ☐ Korean
- ☐ Vietnamese
- ☐ Other Asian
- ☐ Native Hawaiian, Chamorro, Samoan, or Other Pacific Islander

37. Do you currently rent or own your home?

- ☐ Own
- ☐ Rent
- ☐ Occupied without paying monetary rent

38. Which one of these comes closest to your own feelings about your household's income these days?

- ☐ Living comfortably on present income
- ☐ Getting by on present income
- ☐ Finding it difficult on present income
- ☐ Finding it very difficult on present income

39. What is your primary source of health care coverage?

- ☐ A plan purchased through an employer or union (including plans purchased through another person's employer)
- ☐ A plan that you or another family member buys on your own
- ☐ Medicare
- ☐ Medi-Cal
- ☐ TRICARE (formerly CHAMPUS), VA, or Military
- ☐ Alaska Native, Indian Health Service, Tribal Health Services
- ☐ Some other source
- ☐ None (no coverage)
- ☐ Don't know/not sure

40. What is the highest grade or level of schooling you completed?

- ☐ Less than 8 years
- ☐ 8 through 11 years
- ☐ 12 years or completed high school
- ☐ Post high school training other than college (vocational or technical)
- ☐ Some college
- ☐ College graduate
- ☐ Postgraduate

41. What languages do you speak at home? Mark all that apply.

- ☐ English
- ☐ Spanish
- ☐ Cantonese
- ☐ Hmong
- ☐ Korean
- ☐ Mandarin
- ☐ Tagalog
- ☐ Russian
- ☐ Vietnamese
- ☐ Any of the Asian/Indian languages or dialects
- ☐ Other, please specify: ______

42. If you speak a language other than English at home, we are interested in your own opinion of how well you speak English. Would you say you speak English...?

- ☐ Very well
- ☐ Well
- ☐ Not well
- ☐ Not at all
- ☐ Refuse to answer
- ☐ I only speak English
- ☐ Don't know

43. What is your marital status? Mark only one.

- ☐ Married
- ☐ Living as married
- ☐ Divorced
- ☐ Widowed
- ☐ Separated
- ☐ Single, never been married

44. What best describes your current occupational status? Mark only one.

- ☐ Employed
- ☐ Unemployed
- ☐ Homemaker
- ☐ Student
- ☐ Retired
- ☐ Disabled
- ☐ Other, please specify: ____________
- ☐ Don't know

45. What year were you born?

☐☐☐☐ Year

46. Including yourself, how many people live in your household? ☐☐

47. Thinking about members of your family living in this household, what is your combined annual income, meaning the total pretax income from all sources earned in the past year?

- ☐ $0 to $19,999
- ☐ $20,000 to $49,999
- ☐ $50,000 to $74,999
- ☐ $75,000 to $99,999
- ☐ $100,000 to $199,999
- ☐ $200,000 or more
- ☐ Don’t know/not sure

48. Finally, what do you think are the most critical cancer health care issues in your community?

Draft
