# Supplementary material for: Capturing Latino Health Disparities: Lessons from Mail- and Community-Based Population Health Surveys in California
Source: Cancer Res Commun. 2026 Apr 20;6(4):873–83. doi: 10.1158/2767-9764.CRC-25-0540 (PMC13095202; doi:10.1158/2767-9764.CRC-25-0540)
Supplement: Appendix 4 — Mail - Spanish Survey [file crc-25-0540_appendix_4_supps4.pdf]

University of California, Davis Comprehensive Cancer Center
Evaluación de Necesidades de la Comunidad

El propósito de este cuestionario que le tomará alrededor de 10 minutos, es aprender cómo podemos ofrecerle a usted y a otras personas en su comunidad un mejor alcance a programas de prevención, información y control del cáncer.

Instrucciones:
- **Use un bolígrafo de tinta negra o azul para contestar este formulario.**
- **Marque con una ☒ para indicar su respuesta. Si desea cambiar su respuesta, tache el recuadro así: ▇ y marque la respuesta correcta.**

1. ¿Alguna vez ha buscado información sobre temas médicos o de salud de cualquier fuente?
- ☐ Sí
- ☐ No ⟶ PASE A PREGUNTA 5.

2. ¿A quién o dónde acudió primero para obtener información sobre temas médicos o de salud en la ocasión más reciente?
- ☐ Doctor o proveedor de atención médica
- ☐ Familia
- ☐ Otras personas, por ejemplo, amigos, compañeros de trabajo, línea telefónica de avisos
- ☐ Material impreso, por ejemplo, libros, revistas, panfletos
- ☐ Internet, incluyendo redes sociales
- ☐ Otros

3. ¿Hay un sitio de internet o de redes sociales especifico al que le guste acudir para obtener información médica o de salud?
- ☐ Sí
- ☐ No ⟶ PASE A PREGUNTA 5.
- ☐ No sé ⟶ PASE A PREGUNTA 5.

4. ¿Qué plataformas de medios utiliza? Marque todos los que apliquen.
- ☐ Facebook
- ☐ Twitter
- ☐ Instagram
- ☐ LinkedIn
- ☐ Pinterest
- ☐ Snapchat
- ☐ Otro

5. ¿Cuál es el primer hospital que se le viene a la mente cuando piensa en el cuidado del cáncer?

6. Antes de esta encuesta, ¿había oído hablar del UC Davis Comprehensive Cancer Center?
- ☐ Sí
- ☐ No

2517

Detección de cáncer: Las preguntas 7-11 son solo para mujeres.
Hombres, por favor, pasen a pregunta 12.

7. Las próximas preguntas tratan del cáncer del cuello uterino. Una mamografía es una radiografía de cada seno para buscar anomalías que pueden ser en su mayoria benignas (ejemplo fibromas) y en algunos casos, cáncer de mama. ¿Alguna vez se ha hecho una mamografía?

☐ Sí
☐ No ⟶ PASE A PREGUNTA 10.

8. ¿Qué edad tenía cuando le realizaron su primera mamografía? ☐☐

9. ¿Cuánto hace que le realizaron su última mamografía?

☐ En el último año (en cualquier momento, hace 12 meses o menos)
☐ En los últimos 2 años (hace más de 1 año, pero menos de 2 años)
☐ En los últimos 3 años (hace más de 2 años, pero menos de 3 años)
☐ En los últimos 5 años (hace más de 3 años, pero menos de 5 años)
☐ Hace 5 o más años
☐ No sé

10. Un examen de Papanicolaou es un procedimiento para detectar anormalidades en el cuello uterino y permite detectar el cáncer cervical en mujeres. ¿Alguna vez se realizó una citología vaginal?

☐ Sí
☐ No ⟶ PASE A PREGUNTA 12.
☐ No sé ⟶ PASE A PREGUNTA 12.

11. ¿Cuánto tiempo ha pasado desde que tuvo su última prueba de Papanicolau?

☐ En el último año (en cualquier momento, hace 12 meses o menos)
☐ En los últimos 2 años (hace más de 1 año, pero menos de 2 años)
☐ En los últimos 3 años (hace más de 2 años, pero menos de 3 años)
☐ En los últimos 5 años (hace más 3 años, pero menos de 5 años)
☐ Hace 5 o más años

12. La vacuna contra la Hepatitis B se administra en tres dosis separadas y se ha recomendado para todos los recién nacidos desde 1991. ¿Ha recibido alguna vez la serie de 3 dosis de la vacuna contra la Hepatitis B?

☐ Sí, la serie completa de 2 o 3 dosis
☐ Recibí por lo menos una dosis
☐ Ninguna dosis (no vacunado)
☐ No sé

Para los padres de niños de 9 a 17 años. Todos los demás pasen a pregunta 15.

13. ¿Todos sus hijos de 9 a 17 años recibieron una o más dosis de la vacuna contra el virus del papiloma humano (VPH)?

☐ Sí ⟶ PASE A PREGUNTA 15.
☐ No
☐ No sé ⟶ PASE A PREGUNTA 15.

2517

14. ¿Cuál de las siguientes respuestas explica la(s) razón(es) para que sus hijos no sean vacunados contra el VPH? Marque todos los que apliquen.

- ☐ No sé lo suficiente sobre la vacuna contra el VPH
- ☐ El doctor no me lo ha recomendado
- ☐ No pienso que la vacuna es necesaria
- ☐ No sabía que la vacuna podría prevenir el cáncer
- ☐ Factores financieros: demasiado caros o no cubierto por mi seguro
- ☐ Preocupaciones de seguridad con la vacuna
- ☐ Otros, específicamente: ____________

Tabaco

15. ¿Es fumador? Un fumador es alguien que consumió cigarrillos en los últimos 30 días. Sí solía fumar, pero dejó de hacerlo hace más de 30 días, es ex fumador.

- ☐ Fumador → PASE A PREGUNTA 17.
- ☐ Ex Fumador
- ☐ Nunca ha fumado → PASE A PREGUNTA 20.

16. ¿A qué edad fumó por última vez?

☐☐ Años

17. ¿Durante cuántos años ha fumado (o fumó) cigarrillos en total? No cuente los años que no fumo cigarrillos.

☐☐ Años

18. ¿Cuántos cigarrillos fuma (o fumó) al día en promedio? Una cajetilla usualmente tiene 20 cigarrillos.

☐☐ Numero de cigarrillos por día

19. La tomografía computarizada (TC) es un examen que utiliza rayos X para generar imágenes del cuerpo y se utiliza para diagnosticar hemorragias, problemas del corazón y cáncer. ¿Alguna vez ha tenido una tomografía computarizada para la detección del cáncer de pulmón?

- ☐ Sí
- ☐ No

Ejercicio: La siguiente preguntas son sobre sus hábitos generales de ejercicio. El ejercicio incluye caminar, limpieza, trotar, levantar pesas, jugar un deporte o jugar con sus hijos. Se puede hacer en el trabajo, alrededor de la casa, sólo por diversión o como un entrenamiento.

20. En los últimos 7 días, ¿cuántos días realizó por lo menos 20 minutos de ejercicio?

☐ Número de días a la semana

- ☐ No sé

Creencias sobre el Cáncer

Por favor, indique cuánto está usted de acuerdo o en desacuerdo con las siguientes aseveraciones:

	Totalmente de acuerdo	Algo de acuerdo	Algo en desacuerdo	Totalmente en desacuerdo	No sé	Prefiero no responder
**21.** Parece que todo causa cáncer.	☐	☐	☐	☐	☐	☐
**22.** No hay mucho que pueda hacer para reducir sus probabilidades de contraer cáncer.	☐	☐	☐	☐	☐	☐
**23.** Hay muchas recomendaciones diferentes sobre la prevención del cáncer, es difícil saber cuáles seguir.	☐	☐	☐	☐	☐	☐

Acceso a Atención Médica

24. ¿A dónde va para solicitar asesoramiento médico? Marque todos los que apliquen.

- ☐ Clínica o centro de salud
- ☐ Oficina del doctor o HMO
- ☐ Sala de emergencias
- ☐ Departamento ambulatorio del hospital
- ☐ Otro lugar

25. ¿En los últimos 12 meses, alguna vez necesitó ver a un médico, pero no pudo?

- ☐ No, siempre puedo ir a un médico sin problemas → PASE A PREGUNTA 26.

MARQUE TODAS LAS RESPUESTAS QUE DESEE.

- ☐ Sí, porque: No pude obtener una cita
- ☐ Sí, porque: Mi seguro no fue aceptado
- ☐ Sí, porque: Mi seguro no lo cubre
- ☐ Sí, porque: Problemas con el idioma
- ☐ Sí, porque: Problemas con el transporte
- ☐ Sí, porque: Las horas no eran convenientes
- ☐ Sí, porque: No tenía a alguien que cuidara a mis niños
- ☐ Sí, porque: No tuve tiempo
- ☐ Sí, porque: El precio era muy alto
- ☐ Sí, porque: No tenía seguro médico
- ☐ Otros, específicamente: ____________
- ☐ No sé

26. ¿Alguna vez le ha dicho un médico u otro profesional de la salud que tiene o ha tenido cáncer de cualquier tipo?

- ☐ Sí → ¿Qué tipo de cáncer fue? ____________
- ☐ No → PASE A PREGUNTA 30.
- ☐ No sé

27. ¿Cuántos años tenía cuando le diagnosticaron? ☐☐

- ☐ No sé

28. Desde su diagnóstico de cáncer, ¿su doctor le ha recomendado participar en un estudio clínico?

- ☐ Sí, pero escogí no participar
- ☐ Sí, participé en el estudio clínico
- ☐ No, el doctor no recomendó un estudio clínico
- ☐ No sé o no me acuerdo

29. Desde su diagnóstico de cáncer, ¿donó algún muestra, por ejemplo, sangre, células, tejido, tumor etc. para la investigación sobre el cáncer?

- ☐ Sí
- ☐ No, pero sí lo haría
- ☐ No, pero no estoy interesado(a)
- ☐ No sé

Estado de Salud: Las siguientes preguntas son sobre su salud general.

30. ¿Cuál es su altura sin zapatos?

☐ Pies ☐☐ Pulgadas

- ☐ No sé

31. ¿Cuánto pesa sin zapatos?

☐☐☐ Libras

- ☐ No sé

32. ¿Cuanto tiempo ha pasado desde la última vez que visitó a un doctor o proveedor médico para un chequeo de rutina?

- ☐ Hace un año o menos
- ☐ Hace más de 1 año, pero menos de 2
- ☐ Hace más de 2 años, pero menos de 5
- ☐ Hace más de 5 años
- ☐ Nunca
- ☐ No sé

33. Diría usted que, en general, su salud es:

- ☐ Excelente
- ☐ Muy buena
- ☐ Buena
- ☐ No tan buena
- ☐ Mala
- ☐ No sé

2517

Característicos Personales

34. ¿Es hombre o mujer?

- ☐ Hombre
- ☐ Mujer

35. ¿Se considera hispano/a, latino/a o de otro origen hispano, latino o español? Marque todos los que apliquen.

- ☐ Mexicano/a, Mexicano/a Americano/a, Chicano/a
- ☐ Puertorriqueño/a
- ☐ Cubano/a
- ☐ Otro Hispano, Latino/a o de otro origen hispano, latino o español
- ☐ Ninguno de esos

36. ¿A cuál de los siguientes grupos raciales pertenece usted? Marque todos los que apliquen.

- ☐ Blanco/a
- ☐ Negro/a o Afroamericano/a
- ☐ Indio Americano/a o Nativo/a de Alaska
- ☐ Indio Asiático/a
- ☐ Chino/a
- ☐ Filipino/a
- ☐ Hmong
- ☐ Japonés
- ☐ Coreano/a
- ☐ Vietnamita
- ☐ Otro asiático
- ☐ Hawaiano nativo/a, Chamorro, o Samoano/a u otro isleño pacifico

37. ¿En este momento usted renta o es dueño de su casa?

- ☐ Dueño de casa
- ☐ Renta
- ☐ Habita sin pagar renta monetaria

38. ¿Cuál de las siguientes frases se acerca más a sus propios sentimientos sobre los ingresos de su hogar actualmente?

- ☐ Estoy viviendo cómodamente con los ingresos actuales
- ☐ Logro pasar con los ingresos actuales
- ☐ Me resulta difícil mantenerme con los ingresos actuales
- ☐ Me resulta muy difícil mantenerme con los ingresos actuales

39. ¿Cuál es su cubertura de salud principal?

- ☐ Un plan comprado a través de un empleador o Unión/Sindicato (incluyendo los planes comprados a través del empleador de otra persona)
- ☐ Un plan que usted u otro miembro de la familia compra por su cuenta
- ☐ Medicare
- ☐ Medi-Cal
- ☐ Tri-CARE (formalmente CHAMPUS), VA o seguro de militares
- ☐ Nativo de Alaska, Servicio de Salud Indio, Servicio de Salud Tribal
- ☐ Alguna otra fuente
- ☐ Ninguna (sin seguro)
- ☐ No sé/ no estoy seguro/a

40. ¿Cuál es el grado más alto o nivel de escuela que ha completado?

- ☐ Menos de 8 años
- ☐ 8 a 11 años
- ☐ 12 años o escuela secundaria completa
- ☐ Capacitación después de la escuela secundaria que no sea de nivel universitario (tipo técnico o vocacional)
- ☐ Algo de universidad
- ☐ Graduado universitario
- ☐ Estudios de posgrado

41. ¿Que idiomas hablan en su casa? Marque todos los que apliquen.

- ☐ Ingles
- ☐ Español
- ☐ Cantonés
- ☐ Hmong
- ☐ Coreano
- ☐ Mandarin
- ☐ Tagalo
- ☐ Ruso
- ☐ Vietnamita
- ☐ Cualquiera de los idiomas o dialectos asiáticos / indios
- ☐ Otros, específicamente: ______

42. Sí usted habla otro idioma aparte de inglés en su casa, estamos interesados en su propia opinión de qué tan bien habla el idioma de inglés. ¿Diría usted que habla inglés...?

- ☐ Muy bien
- ☐ Bien
- ☐ No muy bien
- ☐ Nada
- ☐ Prefiero no responder
- ☐ Solamente hablo ingles
- ☐ No sé

43. ¿Cuál es su estado civil? Marca solo uno.

- ☐ Casado
- ☐ Viviendo como casados o en una unión libre o de hecho
- ☐ Divorciado
- ☐ Viudo
- ☐ Separado
- ☐ Soltero, nunca se ha casado

44. ¿Cuál es la mejor descripción de su estado laboral? Marca solo uno.

- ☐ Empleado
- ☐ Desempleado
- ☐ Ama de casa
- ☐ Estudiante
- ☐ Retirado
- ☐ Discapacitado
- ☐ Otro, específicamente: ______
- ☐ No sé

45. ¿En qué año nació?

☐☐☐☐ Año

46. Incluyendose a usted mismo, ¿cuántas personas viven en su casa? ☐☐

47. ¿Cuál es el ingreso anual combinado de su hogar antes de impuestos? Para esto, sume los ingresos de todas las fuentes o salarios obtenidos en el ultimo año, por todos los miembros de su familia que comparten la misma casa, sin restar los impuestos.

- ☐ $0 a $19,999
- ☐ $20,000 a $49,999
- ☐ $50,000 a $74,999
- ☐ $75,000 a $99,999
- ☐ $100,000 a $199,999
- ☐ $200,000 o más
- ☐ No sé

48. Finalmente, ¿cuál piensa usted que es el problema más crítico en el cuidado de la salud en su comunidad, especialmente en cuanto a la prevención o manejo del cáncer?

2517
